# Biotechnological Aspects of Microbial Extracellular Electron Transfer

**DOI:** 10.1264/jsme2.ME15028

**Published:** 2015-05-23

**Authors:** Souichiro Kato

**Affiliations:** 1 Bioproduction Research Institute, National Institute of Advanced Industrial Science and Technology (AIST)2–17–2–1 Tsukisamu-Higashi, Toyohira-ku, Sapporo, Hokkaido 062–8517Japan; 2 Division of Applied Bioscience, Graduate School of Agriculture, Hokkaido UniversityKita-9 Nishi-9, Kita-ku, Sapporo, Hokkaido 060–8589Japan; 3 Research Center for Advanced Science and Technology, The University of Tokyo4–6–1 Komaba, Meguro-ku, Tokyo 153–8904Japan

**Keywords:** extracellular electron transfer, microbial electrochemical cells, biocorrosion, bioremediation, electric syntrophy

## Abstract

Extracellular electron transfer (EET) is a type of microbial respiration that enables electron transfer between microbial cells and extracellular solid materials, including naturally-occurring metal compounds and artificial electrodes. Microorganisms harboring EET abilities have received considerable attention for their various biotechnological applications, in addition to their contribution to global energy and material cycles. In this review, current knowledge on microbial EET and its application to diverse biotechnologies, including the bioremediation of toxic metals, recovery of useful metals, biocorrosion, and microbial electrochemical systems (microbial fuel cells and microbial electrosynthesis), were introduced. Two potential biotechnologies based on microbial EET, namely the electrochemical control of microbial metabolism and electrochemical stimulation of microbial symbiotic reactions (electric syntrophy), were also discussed.

Living organisms must constantly acquire energy to continue living and proliferating. They acquire energy through respiration, photosynthesis, or fermentation, all of which are fundamentally based on the oxidation and reduction of chemical compounds and concomitant electron transfer reactions. Most living organisms conduct redox reactions inside of their cells through the incorporation of soluble or gaseous chemical compounds. In contrast, some microorganisms have the ability to acquire energy by transferring electrons to or from extracellular solid materials. Microorganisms that have the ability to utilize solid materials for energy metabolism have a competitive advantage over other organisms, especially in environments in which an electron source or sink availability is limited. Electron exchange reactions between microbial cells and solid materials, which are collectively referred to as “extracellular electron transfer (EET)”, have attracted considerable attention in the fields of microbial physiology, microbial ecology, and biotechnology. This review discussed current knowledge on microbial EET and summarized recent advances in its biotechnological application.

## Mechanisms of microbial EET

**Fig. 1:**
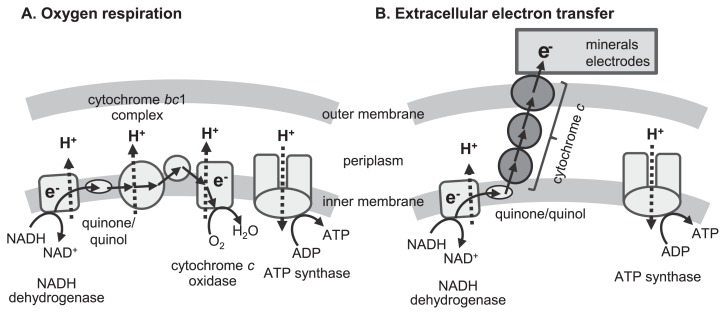
Schematic diagrams of (A) oxygen respiration and (B) microbial extracellular electron transfer.

EET is defined as a microbial metabolic process that enables electron transfer between microbial cells and extracellular solid materials and is a type of microbial respiration. Respiration converts redox potential differences between the oxidation and reduction of chemical compounds into a bioavailable form of energy, generally ATP. Fig. 1A shows a schematic model of oxygen respiration that is found in the majority of living organisms. Most living organisms, including human beings, only utilize a combination of organic compounds and oxygen as an electron donor and acceptor, respectively. On the other hand, energy metabolism with diverse substances acting as the electron acceptors or donors has been detected in microorganisms. Particular microorganisms utilize a wide range of reduced (*e.g.*, H_2_, CH_4_, sulfides, and ammonia) and oxidized (*e.g.*, nitrate, sulfate, and CO_2_) inorganic compounds as electron donors and acceptors, respectively. Other microorganisms have ability to utilize solid materials, such as minerals and electrodes, as substrates for respiration, through a process that is specifically referred to as EET. However, special molecular mechanisms are required for EET reactions because microorganisms cannot incorporate such solid materials into their cells.

Microbial EET has been roughly classified into two different mechanisms: direct and indirect EET. In direct EET, microorganisms attach to solid surfaces, to or from which they directly transfer electrons. The molecular mechanisms for direct EET have been intensively investigated in some model organisms, namely, iron-reducing bacteria *Geobacter sulfurreducens* and *Shewanella oneidensis*, and the ironoxidizing bacterium *Acidithiobacillus ferrooxidans* (8, 86, 99). Redox-active proteins, mainly *c*-type cytochromes, play an important role in direct EET. Electron transfer from inner to outer cell membranes via electron hopping through multiple redox-active proteins connects microbial respiratory chains and external surfaces (Fig. 1B). Furthermore, *G. sulfurreducens* and *S. oneidensis* were previously reported to produce a conductive filamentous apparatus (pili and outer membrane extensions, respectively) that enabled cells to transfer electrons to distantly located solid materials (22, 75, 78).

Redox-active low-molecular compounds, referred to as electron mediators, function as electron carriers between microbial cells and solid materials. Electron mediators reduced (or oxidized) by microorganisms diffuse to solid surfaces and donate (or accept) electrons. Oxidized (or reduced) mediators return to the inside of cells and are again utilized as respiratory substrates. Indirect EET often functions as the dominant type of metabolism in engineered environments (76, 98). Some microorganisms synthesize and excrete small molecules that function as electron mediators, including phenazine compounds and flavin derivatives, which are considered to contribute to indirect EET (60, 76). In contrast, recent studies demonstrated that extracellularly excreted flavin compounds associated with outer membrane cytochromes and accelerated direct EET to solid materials (72, 73). Several reviews have provided more in-depth discussions on the mechanisms of direct and indirect EET (54, 80, 86, 90, 98)

## Biotechnologies based on microbial EET

### Bioremediation of toxic metals

Bioremediation is a technology that involves the use of living organisms to degrade, remove, or detoxify pollutants from contaminated environments. Along with petroleum hydrocarbons and halogenated compounds, toxic metals (and metalloids) are also the targets of bioremediation. In addition to methods based on biosorption and bioaccumulation (20, 41), technologies that focus on the reduction and oxidation of metal compounds via microbial EET have been attracting considerable attention due to their application to the microbiological remediation of toxic metals (47, 55). Bioremediation technologies based on microbial reduction and oxidation have been applied to a number of toxic metal(loid)s. For example, the detoxification of chromium by the reduction of Cr(VI) to Cr(III) (95), the insolubilization of uranium by the reduction of U(VI) to U(III) (30, 53), the abatement of the adsorptive capacity of arsenic compounds by the reduction of arsenate (AsO_4_^3−^) to arsenite (AsO_3_^3−^) (103), and the biomineralization of elemental selenium (Se^0^) via the reduction of selenate (SeO_4_^2−^) and selenite (SeO_3_^2−^) (88) have been reported previously. Furthermore, the detoxification of toxic metals in wastewater by microbial redox reactions in microbial electrochemical systems (discussed below) with the aid of external electrical power, including the reduction of Cr(VI) to Cr(III) by biocathode microbial communities, has recently been undertaken (96).

### Recovery of useful metals

Technologies for the recovery of useful metals have also been developed using microbial reduction and oxidation. Bioleaching is a metal refining technology that elutes useful metals from solid minerals using microbial activities (93). Bioleaching is a long-standing technology and has led to practical applications. The foremost target of bioleaching is the recovery of Cu from copper iron sulfide minerals including chalcopyrite. Acidophilic bacteria such as *Acidithiobacillus* spp., which have the ability to oxidize Fe(II) and sulfur in chalcopyrite by EET, play important roles in bioleaching. In addition to the dissolution effects of acidification through the production of sulfuric acid, the elution of Cu is accelerated due to the oxidation of copper by Fe(III), which is generated by a microbial oxidation reaction. Furthermore, the recovery of precious metals from waste materials (*e.g.*, catalysts, batteries, and electronic parts) using acidophilic iron- and sulfur-oxidizing bacteria has also been investigated (7, 29).

In contrast, many studies have examined the recovery of useful metals based on microbial metal reduction. Microorganisms with EET abilities, including *Shewanella* spp., *Geobacter* spp., and *Desulfovibrio* spp., have been shown to reduce a number of precious metals, including Pt(IV), Pd(II), Rd(III), Ag(I), and Au(III), with similar oxidation-reduction potentials to Fe(III) and Mn(IV) (48). These bacteria reduced these precious metal ions to their respective elemental metals and accumulated metal nanoparticles on their cell surfaces or in their periplasmic spaces (33, 44, 46), making the recovery of precious metals easier. Furthermore, a previous study reported that some metal nanoparticles generated by microbial reduction had high catalytic activities (14). These findings suggest that, in addition to the recovery of precious metals, microbial EET is applicable to the manufacturing technologies of nanomaterials.

### Biocorrosion

The corrosion of iron is an electrochemical process that involves the oxidation of metallic iron (Fe[0]) to Fe(II) and reduction of external electron acceptors. This electron-consuming reaction consists of oxygen reduction under oxic conditions and proton reduction (H_2_ evolution) under anoxic conditions. Since the H_2_ evolution reaction on iron surfaces is typically a particularly slow reaction, iron corrosion in anoxic environments is not considered to be a serious problem. However, iron corrosion has been often reported in anoxic environments, and in most cases, is thought to be mediated by microbial metabolic activities, including microbial EET reactions (3, 24, 94). Dinh *et al.* isolated novel sulfate-reducing bacteria (SRB) and methanogenic archaea from marine sediment with Fe(0) as the sole electron donor (16). These isolates reduced sulfate and produced methane with the concomitant oxidation of Fe(0), respectively, markedly faster than abiotic H_2_ production from Fe(0). The same group demonstrated that the iron-corroding SRB appeared to directly uptake electrons from Fe(0) via EET rather than consuming abiotically generated H_2_ (17, 18, 92). Some strains of methanogenic archaea that have similar iron-corroding activities were also isolated from oil-storage tanks by another research group (65, 91). In addition to SRB and methanogens, a recent study reported that certain acetogenic bacteria appeared to have the ability to induce iron corrosion in anoxic environments (58). Our group isolated acetogenic strains that grew with Fe(0) as the sole electron donor and enhanced iron corrosion, while authentic H_2_- scavenging acetogens did not show such activities (39). Furthermore, Iino *et al.* reported that a non-hydrogenotrophic strain belonging to the phylum *Bacteroidetes* had the ability to induce biocorrosion under nitrate-reducing conditions (28). Although the molecular mechanisms underlying electron transfer from metallic iron to microbial cells currently remain unknown, future investigations on the physiology and ecology of these biocorroding microorganisms may lead to the development of novel technologies to prevent biocorrosion in anoxic environments.

### Microbial fuel cells

In addition to the oxidation and reduction of metal compounds, certain microorganisms have the ability to utilize conductive materials (*e.g.*, graphite electrodes) as the electron donor or acceptor of respiration (6, 23, 43). The ability of microorganisms to transfer electrons to or from electrode materials may be exploited to provide technologies using diverse bioelectrochemical systems, such as microbial fuel cells (MFCs) and microbial electrosynthesis (discussed in the next section). MFCs are one of the most extensively investigated bioelectrochemical systems in which chemical energy stored in a substrate (*e.g.*, waste organics) is directly converted into electrical energy through microbial metabolism (49, 50, 97). MFCs have advantages over fuel cells: 1) diverse organic matter including waste compounds may be utilized as fuel, 2) MFCs operate stably, even at ordinary temperatures, and 3) the electrode catalysts, namely microorganisms, are cheap and self-propagating. Although the performance of MFC has been largely improved by modifications to reactor configurations, electrode materials, and cathode catalysts (52, 64, 105), the electricity output of MFC is markedly lower than that of chemical fuel cells. Hence, most studies on MFCs target the development of low-energy intensive wastewater treatment systems. Further practical research to solve some critical issues, including enlargement of the reactor scale, long-term durability, and the relatively high cost of electrode materials, is required for the commercialization of MFC-based wastewater treatment systems (25, 51, 52). In addition to wastewater treatment systems, the application of MFCs to portable batteries (27, 79), remote batteries in marine and lake sediments (4), and electricity generation in plant rhizospheres (*e.g.*, rice paddy fields) (15, 31) has also been investigated.

### Microbial electrosynthesis

Microbial electrosynthesis is a biotechnology based on microbial energy conversion from electricity to chemical fuels (56, 77, 82). The utilization of microbial CO_2_ fixation activities for the synthesis of chemical fuels has advantages over technologies based on inorganic catalysts because the production of multi-carbon compounds from CO_2_ with inorganic catalysts is extremely difficult. Acetogenic bacteria are expected as the “biocatalysts” to produce chemical fuels from CO_2_ with the input of electrical energies. Nevin *et al.* reported that some acetogenic bacteria, which generally acquire energy through the generation of acetate from CO_2_ with H_2_ as the electron donor, had the ability to convert electrical energy into multi-carbon chemicals (acetate and a small amount of 2-oxobutyrate) under conditions with poised electrodes as the sole electron donor (68, 69). In addition to acetogenic bacteria, the microbial production of H_2_ and CH_4_ via the reduction of protons and CO_2_, respectively, with cathode electrodes as the electron sources also represents a prospective new biological energy conversion system (11, 84). While the molecular mechanisms of electron transfer from electrodes to microbial cells remain largely unknown (81), many research projects to improve microbial electrosynthesis, including the metabolic engineering of acetogenic bacteria (2) and improvements to cathode materials (104), are now ongoing.

### Electrochemical control of microbial metabolism

Shifts in the cellular redox balance largely affect microbial gene expression and metabolism. The artificial control of microbial metabolism by electrochemical alterations to the cellular redox balance has been extensively investigated since the 1970s. The electrochemical control of microbial metabolism has considerable advantages over other methodologies because the continuous and/or intermittent adjustment of microbial metabolism without gene modifications or the supplementation of chemical inducers is theoretically possible. Most of these studies utilized artificial mediator compounds, including neutral red and methyl viologen, to achieve electrical connections between electrodes and microbial cells, and demonstrated that it was possible to enhance the production of fuel compounds and amino acids by supplying reducing equivalents from external electrical power sources to fermentative microorganisms (26, 42, 74). However, the practical uses of such artificial mediators were hampered by their cost, stability, and cytotoxicity.

Recent studies proposed two different methodologies to overcome mediator issues. The first one is the electrical control of metabolism by microorganisms that innately possess EET abilities. Previous studies reported that microorganisms with EET abilities, including *Geobacter* spp. and *Shewanella* spp., altered their gene expression and metabolic fluxes depending on shifts in electrode potentials (61, 62). Flynn *et al.* showed that the conversion of glycerol to ethanol by *S. oneidensis* was accelerated by altering the anode potential (19). Steinbusch *et al.* demonstrated that the production of ethanol via the reduction of acetate by microbial communities enriched on anodic electrodes of MFC may also be enhanced by electrochemical control (87).

Another methodology is the development of biocompatible electron mediators. The utilization of mediator compounds with low cytotoxicity enables the electrochemical control of microorganisms known to have abilities in the bioproduction of useful chemicals. Coman *et al.* developed flexible osmium redox polymers to achieve efficient electric communication between electrodes and diverse microorganisms, including the Gram-positive bacterium *Bacillus subtilis* (12). Our research group newly synthesized electron-mediating co-polymers consisting of an amphipathic phospholipid-like domain and redox-active vinylferrocene domain, which enabled electron transfer between electrodes and diverse microorganisms, including *Escherichia coli* and *Lactobacilli*, with low cytotoxicity (70). Our group also reported the enhancement of polyhydroxybutyrate production by *Ralstonia eutropha* (71) and electrochemical control of circadian rhythms of *Synechococcus elongates* (57) using the biocompatible redox polymers. The further development of new electron mediators with low cost, low cytotoxicity, and superior electron transfer abilities will aid in the practical application of EET-based enhancements to the bioproduction of useful chemicals.

### Electric syntrophy: electrochemical stimulation of microbial symbiotic reactions

**Fig. 2:**
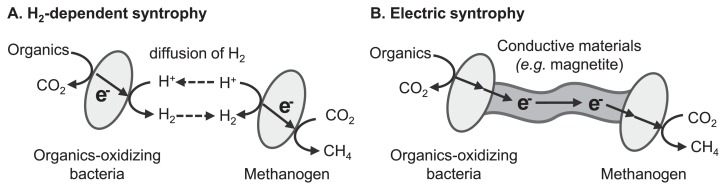
Schematic diagrams of (A) H_2_-dependent syntrophic methanogenesis and (B) methanogenesis based on electric syntrophy.

Some important microbiological processes proceed via the cooperation of multiple microbial species through energy exchanges. This type of microbial symbiosis is specifically termed syntrophy. Small molecules such as organics, nitrogen and sulfur compounds, and H_2_ generally function as energy carriers (Fig. 2A). In addition, interspecies energy exchange is mediated by electric currents flowing through conductive solid materials in the recently found syntrophic interaction, which is specifically termed electric syntrophy or direct interspecies electron transfer (Fig. 2B). Electric syntrophy requires “electrical wires” that connect the metabolism of two different microbial cells. Summers *et al.* demonstrated that conductive pili produced by the microorganisms themselves functioned as wires that electrically connected the respiratory metabolism of *G. metallireducens* and *G. sulfurreducens* (89). On the other hand, our group expected naturally occurring conductive iron oxide minerals (*e.g.*, magnetite) to function as wires for electric syntrophy, based on the knowledge that some electricity-generating bacteria have the ability to exchange electrons with conductive iron oxide minerals (34, 38, 67). Our group demonstrated electric syntrophy based on conductive iron oxides using a model microbial consortium consisting of two EET-harboring bacterial species, namely *G. sulfurreducens* and *Thiobacillus denitrificans* (36).

Electric syntrophy has received considerable attention for its application to various biotechnologies, including the enhancement of known microbial syntrophic processes and design of novel microbial syntrophic reactions. One of the most intensively studied processes is the enhancement of microbial methanogenesis, which has already been utilized for energy-saving waste (water) treatment processes. Microbial methanogenesis from organic compounds requires the cooperation of multiple microbial species, in which the syntrophic reactions of organic acid-oxidizing bacteria and hydrogenotrophic methanogenic archaea often become the rate-limiting step (35, 85). This type of syntrophic reaction is generally mediated by interspecies electron transfer with H_2_ as the electron carrier (Fig. 2A). Our group demonstrated that the supplementation of conductive iron oxide particles induced electric syntrophy and enhanced methanogenic reactions in microbial communities derived from rice paddy field soil (37) and a thermophilic methane-fermenting reactor (102). Other research groups reported enhancements to methanogenesis through electric syntrophy via conductive iron oxides (13), graphite (10, 45), biochar (9), and microbial nanowires (66, 83). Furthermore, Aulenta *et al.* showed that conductive iron oxides accelerated the microbial reductive dechlorination of trichloroethene by promoting interspecies electron transfer processes (1), suggesting that the stimulation of microbial syntrophy has potential in the field of bioremediation. Further investigations on the microbial physiology of electric syntrophy and development of low-cost materials with high conductivity and high biocompatibility will shed light on the applicational use of electric syntrophy.

## Concluding remarks

Microbial EET and its possible applications were summarized in this review. Microbial EET has been intensively studied in limited model species belonging to *Proteobacteria*, namely, species in the genus *Geobacter*, *Shewanella*, and *Acidithiobacillus*. However, recent studies demonstrated that phylogenetically more diverse microorganisms, including Gram-positive bacteria (*e.g.*, *Firmicutes*), *Bacteroidetes*, *Cloroflexi*, and *Archaea*, exhibited EET abilities (5, 21, 40, 59, 100, 101). Furthermore, a genome sequence analysis on diverse microbial species and metagenomics analysis on environmental samples revealed that various microorganisms, including methane-oxidizing bacteria (32) and microorganisms in anaerobic methane-oxidizing consortia (63), encoded genes for the outer membrane *c*-type cytochromes required for EET reactions. These findings indicate that EET is a microbial trait that is more widespread among diverse microbial clades than was initially thought. Research on microbial EET will be of great importance for understanding global materials and energy cycles, in addition to contributing to the development of new biotechnologies.
